# Correlation between orbital imaging features of thyroid-associated ophthalmopathy and pupillary light reflex measurement

**DOI:** 10.3389/fmed.2025.1552729

**Published:** 2025-03-21

**Authors:** Yongran Li, Ziao Zhu, Yanling Lu, Qihui Lin, Miaozhi Liu, Zeyi Li

**Affiliations:** ^1^Joint Shantou International Eye Center of Shantou University and the Chinese University of Hong Kong, Shantou, China; ^2^Shunde Aier Eye Hospital, Foshan, China; ^3^Department of Radiology, Second Affiliated Hospital of Shantou University Medical College, Shantou, China

**Keywords:** extraocular muscles, magnetic resonance imaging, pupillometer, pupillary light relax, thyroid-associated ophthalmopathy

## Abstract

**Purpose:**

This study aimed to assess the factors associated with quantitative pupillary light reflex analysis and orbital magnetic resonance imaging (MRI) indicators in thyroid-associated ophthalmopathy (TAO) patients with different TAO severities, and their diagnostic significance of dysthyroid optic neuropathy (DON).

**Methods:**

A retrospective cross-sectional analysis was conducted on 57 patients with TAO, involving 109 orbits. Using the EUGOGO severity grading system, patients were categorized into three groups: Mild TAO (45 orbits), Moderate-to-Severe TAO (48 orbits), and DON (16 orbits). All participants underwent comprehensive ophthalmological assessments, pupillary light reflex analysis using the RAPDx device (Konan Medical), and MRI imaging (GE 3.0 Signa Creator, GE Medical Systems). MRI measurements included orbital bone wall area, extraocular muscle area, and proptosis. Differences in clinical characteristics, pupillary function indicators, and MRI-derived indicators were analyzed using Generalized Estimating Equations (GEE). Correlations and trends between Latency Onset of Constriction (LOC) and MRI indicators were assessed through Pearson multivariate analysis and linear regression models. The diagnostic value of LOC and the Volume of the Medial Orbital Wall (VMW) for diagnosing DON was further evaluated using Receiver Operating Characteristic (ROC) curve analysis.

**Results:**

The results revealed that LOC was significantly prolonged in the DON group compared to both the Mild TAO and Moderate-to-Severe TAO groups (*p* < 0.05 for both). LOC demonstrated strong positive correlations with Inferior Orbital Nerve Signal Loss (IONSL) (*r* = 0.494, *p* < 0.001), Proptosis (*r* = 0.448, p < 0.001), and Medial Rectus Area (MRA) (*r* = 0.428, *p* < 0.001). Multivariate binary logistic regression analysis identified LOC and VMW as independent predictors of DON. A predictive model combining LOC and VMW showed excellent diagnostic performance, with an Area Under the Curve (AUC) of 0.886 (*p* < 0.001), sensitivity of 90.5%, and specificity of 82.4%.

**Conclusion:**

These findings underscore the critical roles of pupillary light reflex analysis and MRI in diagnosing and evaluating TAO. The significant correlations of LOC with IONSL, Proptosis, and MRA, along with its strong predictive value alongside VMW, highlight their utility as reliable diagnostic markers for DON.

## 1 Background

Thyroid-associated ophthalmopathy (TAO) is an organ-specific autoimmune condition linked to autoimmune thyroid disorders, representing approximately 20% of orbital diseases in adults (1). Severe manifestations of TAO can lead to upper eyelid retraction, extraocular muscle inflammation or fibrosis, and more critically, dysthyroid optic neuropathy (DON) (2, 3), which can cause color vision abnormalities, visual field defects, substantial visual acuity deterioration, and optic nerve atrophy.

Currently, the diagnosis of DON involves a range of diagnostic tools, including visual acuity testing, visual evoked potentials (VEP), visual field testing, color vision testing, computed tomography (CT) (4), magnetic resonance imaging (MRI), and relative afferent pupillary defect (RAPD) (5). However, a universally accepted gold standard for diagnosing DON remains unavailable.

MRI facilitates multiparametric imaging, enabling the measurement of various orbital tissues and the orbital apex system (6). Key indicators include the angle between the rectus muscles and the optic nerve, the orbital apex crowding score, and exophthalmos. The prevailing hypothesis regarding DON pathogenesis posits that it is primarily driven by orbital apex tissue crowding, which induces microcirculatory disturbances and compresses the optic nerve (7).

Callahan et al. (5) demonstrated the diagnostic significance of RAPD in DON. However, most existing literature on pupillary light reflexes relies on qualitative assessments based on clinical experience and flashlight tests. The RAPDx pupillometer offers a more precise method for quantifying dynamic pupil changes, enabling accurate detection and comparison of subtle variations that are not perceptible to the naked eye (8–10).

This study represents the first application of quantitative pupillary light reflex analysis using RAPDx in conjunction with MRI-based measurements of intraorbital tissue relationships to assist in the diagnosis of DON.

## 2 Methods

### 2.1 Study population

This retrospective study was conducted on patients with TAO who visited the Joint Shantou International Eye Center (JSIEC) from May 2021 to March 2023. A total of 57 patients, including 28 males and 29 females, were enrolled, with 109 eyes examined. All TAO diagnoses were made through comprehensive clinical and imaging assessments. The study adhered to the principles set forth in the Declaration of Helsinki and received approval from the Ethics and Academic Committee of the JSIEC. Data and materials collected during the study were handled with strict confidentiality.

### 2.2 Diagnostic criteria and exclusion criteria

Each participant underwent detailed ophthalmic evaluations performed by experienced optometrists, ophthalmologists, and medical technicians. The diagnostic criteria followed Bartley’s standards and incorporated the 2021 EUGOGO European Thyroid Eye Disease Guidelines and the 2022 Chinese Guidelines for the Diagnosis and Treatment of Thyroid Eye Disease (11, 12). In this study, patients with very severe TAO exclusively included patients with DON. The diagnosis of DON was based on the joint consensus of ATE/ETA (13), with patients meeting the following criteria diagnosed with DON: Presence of at least one of the following clinical or imaging findings: (1) Ishihara color plates: correct identification of fewer than 40% of plates; (2) best-corrected logMAR visual acuity ≥0.3; (3) abnormal pattern visual evoked potential (PVEP) results: moderate reduction or worse in P100 amplitude; (4) visual field loss: mean defect value ≥5 dB; (5) clinical signs: optic disc edema or atrophy.

Exclusion criteria were as follows: (1) patients with very severe TAO exhibiting severe exposure keratitis; (2) patients who received high-dose steroid pulses, immunosuppressive therapy, or radiation therapy within the past 6 months, or those with a history of orbital decompression surgery; (3) patients under the age of 20; (4) patients with a history of ocular diseases affecting vision, such as optic neuropathy, retinal disease, glaucoma, severe cataracts, congenital ocular abnormalities; (5) patients with a history of ocular trauma or intraocular surgery; (6) patients with a history of intracranial infection, Parkinson’s syndrome, or other neurological disorders.

### 2.3 Research methods

#### 2.3.1 Pupillary function analysis

**Figure 1:**
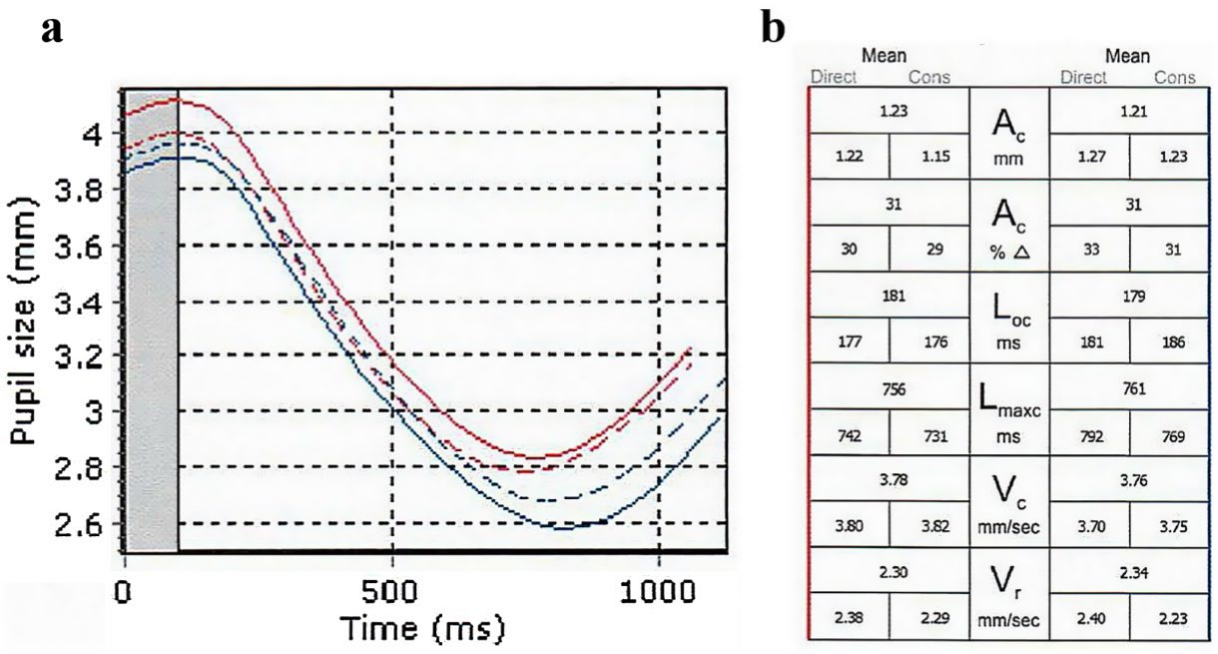
Pupillometric analysis report results for TAO patients. **(A)** Pupillary response curves after light stimulation. Red indicates the right eye, and blue indicates the left eye. Solid lines indicate direct light reflex, while dashed lines indicate indirect light reflex. The initial pupil diameter is read from the graph. **(B)** Detailed data for six pairs of indicators, representing direct light reflex, indirect light reflex, and mean light reflex.

Pupillary function was analyzed using the RAPDx (Konan Medical, Irvine, California, United States). All participants were dark-adapted for at least 2 min prior to the examination, which was conducted in a quiet, dark environment. The examinations were performed between 8:00 a.m. and 12:00 p.m. The RAPDx light stimulation indicators were configured as follows: background illumination of 0.5 lx, a 30-degree visual field, white stimulus illumination of 30.9 lx, a stimulus duration of 0.2 s, an inter-stimulus interval of 2.1 s, and a total of 18 stimuli. The RAPDx alternately delivered light stimuli to each eye, inducing pupillary reflexes while measuring and recording direct light reflex, indirect light reflex, and mean light reflex data for each eye. For the purposes of this study, only the direct light reflex data were utilized, which included the following indicators: Pupil diameter (PD), which represents resting pupil size; Amplitude of constriction (AC);Response amplitude (AC%), defined as the amplitude of constriction as a percentage of resting pupil size; Latency onset of constriction (LOC), the latency of onset of constriction from the beginning of the stimulus; Latency of maximum constriction (LMC), the latency of peak constriction from the beginning of the stimulus; Velocity of peak constriction (VC); and Velocity of peak re-dilation (VR) (Figure 1).

#### 2.3.2 Orbital MRI indicator measurements

In this study, a 3.0 T magnetic resonance system (GE 3.0 Signa Creator, GE Medical Systems, United States) was used to perform orbital magnetic resonance imaging on patients. The imaging sequences used: (1) Axial T1-weighted imaging (T1WI): 3 mm slice thickness, repetition time (TR): 400–600 ms, echo time (TE): 7–10 ms; (2) Axial T2-weighted imaging with fat suppression (T2WI FS) in transverse plane: 3 mm slice thickness, TR: 2000–3,000 ms, TE: 65–70 ms; (3) Sagittal T2-weighted imaging with fat suppression (T2WI FS): 3 mm slice thickness, TR: 2000–3,000 ms, TE: 65–70 ms.

**Figure 2:**
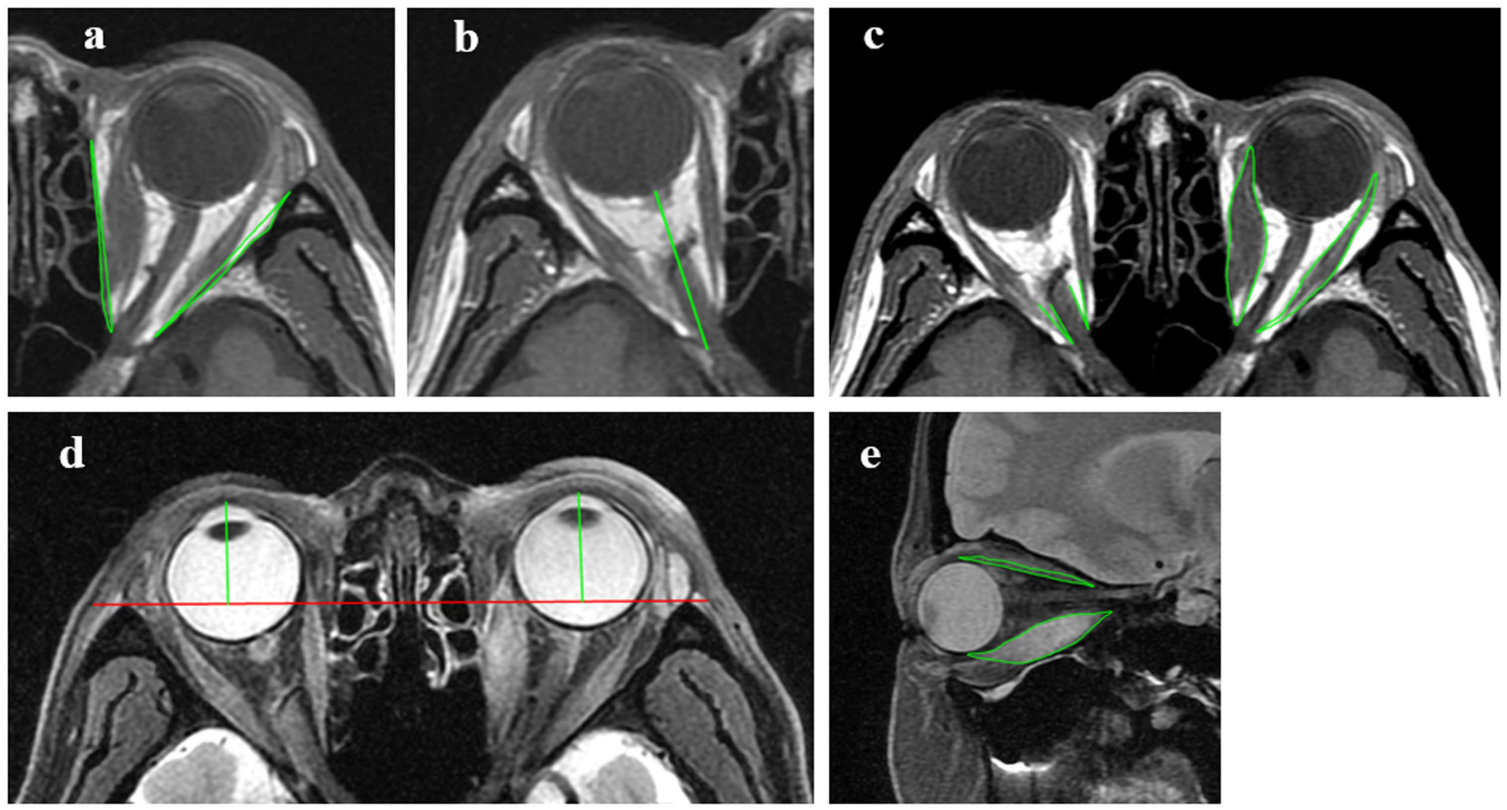
Measurements of MRI indicators. **(A)** Axial T1WI, measurements of the volume of the medial orbital wall and the volume of the lateral orbital wall. **(B)** Axial T1WI, measurements of the intraorbital optic nerve stretching length. **(C)** Axial T1WI, measurements of the medial and lateral rectus area, the angle between the medial rectus and optic nerve and the angle between the lateral rectus and optic nerve. **(D)** Axial T2W2 FS, measurements of proptosis. **(E)** Sagittal T2WI FS, measurements of the superior and inferior rectus area.

To minimize errors due to extraocular muscle contraction and eye position deviation, patients were instructed to look straight ahead and slightly close their eyes. The following indicators were measured using the Carestream GCRIS 2.1 workstation (Carestream, United States): (1) Volume of the Medial Orbital Wall (VMW): Defined by the area formed by connecting the lateral apex of the lacrimal bone to the medial edge of the optic nerve canal and the medial bone wall. The total area is considered positive if it protrudes laterally and negative if it is indented medially; (2) Volume of the Lateral Orbital Wall (VLW): Defined by the area formed by connecting the medial apex of the zygomatic bone to the lateral edge of the superior orbital fissure and the lateral bone wall; (3) Intraorbital Optic Nerve Stretching Length (IONSL): The distance from the posterior globe origin of the optic nerve to the orbital apex; (4) Proptosis: Measured as the perpendicular distance from the corneal apex to the inter-zygomatic line, which is defined as the line connecting the most anterior points of the bilateral zygomatic bones; (5) Medial Rectus Area (MRA) and Lateral Rectus Area (LRA): The areas of the medial and lateral rectus muscles; (6) Angle Between the Medial Rectus and Optic Nerve (AMR-ON): The angle between the medial rectus muscle and the optic nerve; (7) Angle Between the Lateral Rectus and Optic Nerve (ALR-ON): The angle between the lateral rectus muscle and the optic nerve; (8) Superior Rectus Area (SRA) and Inferior Rectus Area (IRA): The areas of the superior and inferior rectus muscles (Figure 2).

### 2.4 Statistical methods

Patient data were processed using Microsoft Excel, while statistical analyses were conducted with SPSS 22.0 and GraphPad Prism. Measurement data were assessed for normality and confirmed to follow a normal distribution, with results expressed as mean ± standard deviation. Gender ratio differences between groups were evaluated using the χ^2^-test. Group differences in clinical indicators, pupillary function indicators, and MRI indicators were analyzed through Generalized Estimating Equations (GEE). Correlation and trend analyses between LOC and MRI indicators were performed using Pearson multivariate analysis and linear regression models. Multivariate binary logistic regression analysis was applied to indicators such as LOC, MRA, and LRA to determine independent predictors of DON. The diagnostic value of LOC and VMW for DON was assessed through Receiver Operating Characteristic (ROC) curve analysis. Statistical significance was defined as a *p*-value <0.05.

## 3 Results

### 3.1 Basic demographic data

A total of 57 patients were included in the study, consisting of 28 males and 29 females, with 109 eyes evaluated. Among these, 45 eyes were classified in the mild TAO group, 48 in the moderate-to-severe TAO group, and 16 in the DON group (Table 1). No significant differences were observed in age (*p* = 0.480) or sex distribution (*p* = 0.510) across the three groups. Visual acuity exhibited significant differences in all pairwise group comparisons (0.01 ± 0.02 vs. 0.07 ± 0.09, *p* < 0.001; 0.01 ± 0.02 vs. 0.22 ± 0.14, *p* < 0.001; 0.07 ± 0.09 vs. 0.22 ± 0.14, *p* < 0.001). Intraocular pressure (IOP) differed significantly only between the mild TAO and moderate-to-severe TAO groups (18.73 ± 3.17 vs. 20.75 ± 5.97, *p* = 0.04).

**Table 1:** Baseline data.

	Mild TAO	Moderate-to-severe TAO	DON	P1	P2	P3
Eyes	45	48	16	–	–	–
Age(years)	42.94 ± 9.94	43.22 ± 11.87	53.43 ± 12.55	–	–	–
Sex(male/female)	23/22	22/26	10/6	–	–	–
logMAR visual acuity	0.01 ± 0.02	0.07 ± 0.09	0.22 ± 0.14	<0.001^***^	<0.001^***^	<0.001^***^
IOP(mmHg)	18.73 ± 3.17	20.48 ± 6.14	20.75 ± 5.97	0.040^*^	0.162	0.799

*p < 0.05, **p < 0.01, ***p < 0.001. P1, the Mild TAO group vs. the Moderate-to-Severe TAO group; P2, the Mild TAO group vs. the DON group; P3, the Moderate-to-Severe TAO group vs. the DON group; Age, the Mild TAO group vs. the Moderate-to-Severe TAO group vs. the DON group; *p* = 0.480. Sex: the Mild TAO group vs. the Moderate-to-Severe TAO group vs. the DON group, *p* = 0.510.

### 3.2 Differences in pupillary function indicators and MRI indicators among different groups

Regarding pupillary function indicators (Table 2), LOC exhibited significant differences between the mild TAO group and the DON group, as well as between the moderate-to-severe TAO group and the DON group (*p* < 0.001 and *p* = 0.04, respectively). LMC showed a significant difference between the mild TAO group and the DON group (*p* = 0.004).

**Table 2:** Comparison of pupillometric and MRI indicators across different severity groups of TAO.

	Mild TAO	Moderate-to-severe TAO	DON	P1	P2	P3
RAPDx indicators						
PD(mm)	4.57 ± 0.84	4.94 ± 0.73	4.95 ± 0.81	0.287	0.869	0.305
AC(mm)	1.4 ± 0.37	1.45 ± 0.28	1.33 ± 0.33	0.829	0.504	0.496
AC%	30.44 ± 4.83	29.5 ± 4.65	27 ± 4.35	0.320	0.210	0.119
LOC(ms)	164.53 ± 12.85	168.33 ± 20.66	188.25 ± 18.81	0.410	<0.001^***^	0.040^*^
LMC(ms)	697.38 ± 49.18	706.73 ± 52.07	731.63 ± 71.38	0.580	0.004^**^	0.189
VC(mm/s)	4.58 ± 1.13	4.75 ± 0.83	4.4 ± 0.91	0.173	0.842	0.639
VR(mm/s)	2.57 ± 0.54	2.7 ± 0.71	2.46 ± 0.55	0.127	0.917	0.167
MRI indicators						
VMW(cm^2^)	0.08 ± 0.34	0.14 ± 0.31	−0.26 ± 0.27	0.732	<0.001^***^	0.023^*^
VLW(cm^2^)	0.62 ± 0.23	0.57 ± 0.19	0.7 ± 0.26	0.561	0.312	0.088
IONSL(cm)	3.11 ± 0.17	3.32 ± 0.27	3.57 ± 0.4	0.001^**^	<0.001^***^	0.357
Proptosis(cm)	1.83 ± 0.21	2.11 ± 0.28	2.25 ± 0.42	<0.001^***^	<0.001^***^	0.826
MRA(cm^2^)	0.96 ± 0.29	1.16 ± 0.37	2.06 ± 0.7	0.010^*^	0.468	0.552
LRA(cm^2^)	0.39 ± 0.17	0.46 ± 0.21	0.84 ± 0.38	0.003^**^	<0.001^***^	0.004^**^
SRA(cm^2^)	0.72 ± 0.24	0.87 ± 0.3	1.3 ± 0.55	0.010^**^	<0.001^***^	0.145
IRA(cm^2^)	1.25 ± 0.39	1.51 ± 0.39	2.03 ± 0.66	<0.001^***^	<0.001^***^	0.003^**^
AMR-ON(°)	15.95 ± 6.19	11.47 ± 5.48	2.69 ± 5.08	<0.001^***^	<0.001^***^	<0.001^***^
ALR-ON(°)	21.47 ± 5.89	18.23 ± 8.1	7.14 ± 8.24	0.080	<0.001^***^	0.001^**^

*P < 0.05, **P < 0.01, ***P < 0.001. P1 denotes the comparison between Mild TAO group and Moderate to Severe TAO group; P2 denotes the comparison between Mild TAO group and DON group; P3 denotes the comparison between Moderate to Severe TAO group and DON group. PD, Pupil diameter; AC, Amplitude of constriction; AC%, Response amplitude; LOC, Latency onset of constriction; LMC, Latency of maximum constriction; VC, Velocity of peak constriction; VR, Velocity peak re-dilation; VMW, The volume of the medial orbital wall; VLW, The volume of the lateral orbital wall; IONSL, The intraorbital optic nerve stretching length; MRA, Medial rectus area; LRA, Lateral rectus area; SRA, Superior rectus area; IRA, Inferior rectus area; AMR-ON, The angle between the medial rectus and optic nerve; ALR-ON, The angle between the lateral rectus and optic nerve.

For MRI indicators related to bony structures, VMW demonstrated significant differences between the mild TAO group and the DON group, as well as between the moderate-to-severe TAO group and the DON group (*p* < 0.001 and *p* = 0.023, respectively).

In the evaluation of soft tissues, IONSL revealed significant differences between the mild TAO group and the moderate-to-severe TAO group, as well as between the mild TAO group and the DON group (*p* = 0.001 and *p* < 0.001, respectively). Similarly, proptosis showed significant differences between the mild TAO group and the moderate-to-severe TAO group, as well as between the mild TAO group and the DON group (*p* = 0.001 and *p* < 0.001, respectively).

In the comparison of extraocular muscle indicators, MRA exhibited a significant difference between the mild TAO group and the moderate-to-severe TAO group (*p* = 0.001). LRA showed significant differences across all pairwise comparisons (*p* = 0.003, *p* < 0.001, and *p* = 0.004, respectively). IRA displayed significant differences between the mild TAO group and the moderate-to-severe TAO group, as well as between the mild TAO group and the DON group (*p* = 0.01 and *p* < 0.001, respectively). SRA showed significant differences across all pairwise comparisons (*p* < 0.001, *p* < 0.001, and *p* = 0.003, respectively).

For angular measurements between extraocular muscles and the optic nerve, AMR-ON showed significant differences across all pairwise comparisons (*p* < 0.001 for all). ALR-ON exhibited significant differences between the mild TAO group and the DON group, as well as between the moderate-to-severe TAO group and the DON group (*p* < 0.001 and *p* = 0.001, respectively).

### 3.3 The correlation between LOC and clinical and MRI indicators

**Figure 3:**
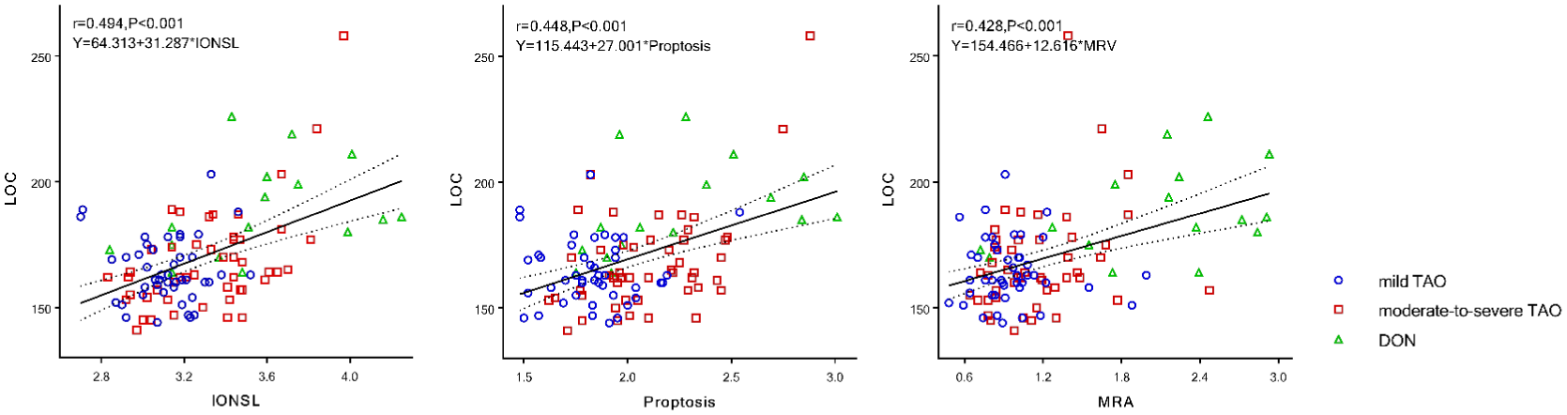
Pearson correlation between LOC, IONSL, proptosis, and MRA. LOC, latency onset of constriction; IONSL, the intraorbital optic nerve stretching length; MRA, the medial rectus area.

Pearson correlation analysis revealed that visual acuity (*r* = 0.253, *p* < 0.001), IONSL (*r* = 0.494, *p* < 0.001), proptosis (*r* = 0.448, *p* < 0.001), MRA (*r* = 0.428, *p* < 0.001), LRA (*r* = 0.365, *p* < 0.001), SRA (*r* = 0.274, *p* = 0.004), and IRA (*r* = 0.347, *p* < 0.001) positively correlated with LOC. AMR-ON (*r* = −0.295, *p* = 0.002) and ALR-ON (*r* = −0.253, *p* = 0.008) negatively correlated with LOC, with statistically significant differences observed. Among these, IONSL, proptosis, and MRA demonstrated moderate correlations with LOC (Figure 3; Table 3).

**Table 3:** Correlation analysis of LOC with clinical and MRI indicators.

	*r*	*P*-value		*r*	*P*-value
logMAR visual acuity	0.353	<0.001^***^	MRA(cm^2^)	0.428	<0.001^***^
IOP(mmHg)	0.104	0.284	LRA(cm^2^)	0.365	<0.001^***^
VMW(cm^2^)	−0.113	0.347	SRA(cm^2^)	0.274	0.004^**^
VLW(cm^2^)	−0.037	0.702	IRA(cm^2^)	0.347	<0.001^***^
ONSL(cm)	0.494	<0.001^***^	AMR-ON(°)	−0.295	0.002^**^
Proptosis(cm)	0.448	<0.001^***^	ALR-ON(°)	−0.253	0.008^**^

*P < 0.05, **P < 0.01, ***P < 0.001. VMW, The volume of the medial orbital wall; VLW, the volume of the lateral orbital wall; IONSL, The intraorbital optic nerve stretching length; MRA, Medial rectus area; LRA, Lateral rectus area; SRA, Superior rectus area; IRA, Inferior rectus area; AMR-ON, The angle between the medial rectus and optic nerve; ALR-ON, The angle between the lateral rectus and optic nerve.

### 3.4 Multivariate binary logistic regression analysis of multiple indicators for the occurrence of DON

Significant indicators differentiating the mild TAO and moderate-to-severe TAO groups from the DON group were analyzed using multivariate binary logistic regression to identify factors associated with the occurrence of DON. LOC (OR = 1.038, *p* = 0.015) and VMW (OR = 0.078, *p* = 0.028) were determined to be independent influencing factors. An increase in LOC and a decrease in VMW were associated with a greater likelihood of DON development (Table 4).

**Table 4:** Multivariate binary logistic regression analysis of multiple indicators for the prediction of DON.

	OR -value	95%CI	Wald χ^2^	*P*-value
LOC(ms)	1.038	(1.007, 1.069)	5.885	0.015^*^
VMW(cm^2^)	0.078	(0.008, 0.754)	4.853	0.028^*^
LRA(cm^2^)	0.641	(0.061, 6.79)	0.136	0.712
IRA(cm^2^)	2.312	(0.241, 22.193)	0.528	0.468
AMR-ON(°)	0.880	(0.743, 1.041)	2.221	0.136
ALR-ON(°)	0.888	(0.763, 1.032)	2.388	0.122

*P < 0.05, **P < 0.01, ***P < 0.001. LOC, Latency onset of constriction; VMW, The volume of the medial orbital wall; LRA, Lateral rectus area; IRA, Inferior rectus area; AMR-ON, The angle between the medial rectus and optic nerve; ALR-ON, The angle between the lateral rectus and optic nerve.

### 3.5 Diagnostic value of LOC and VMW for DON

**Figure 4:**
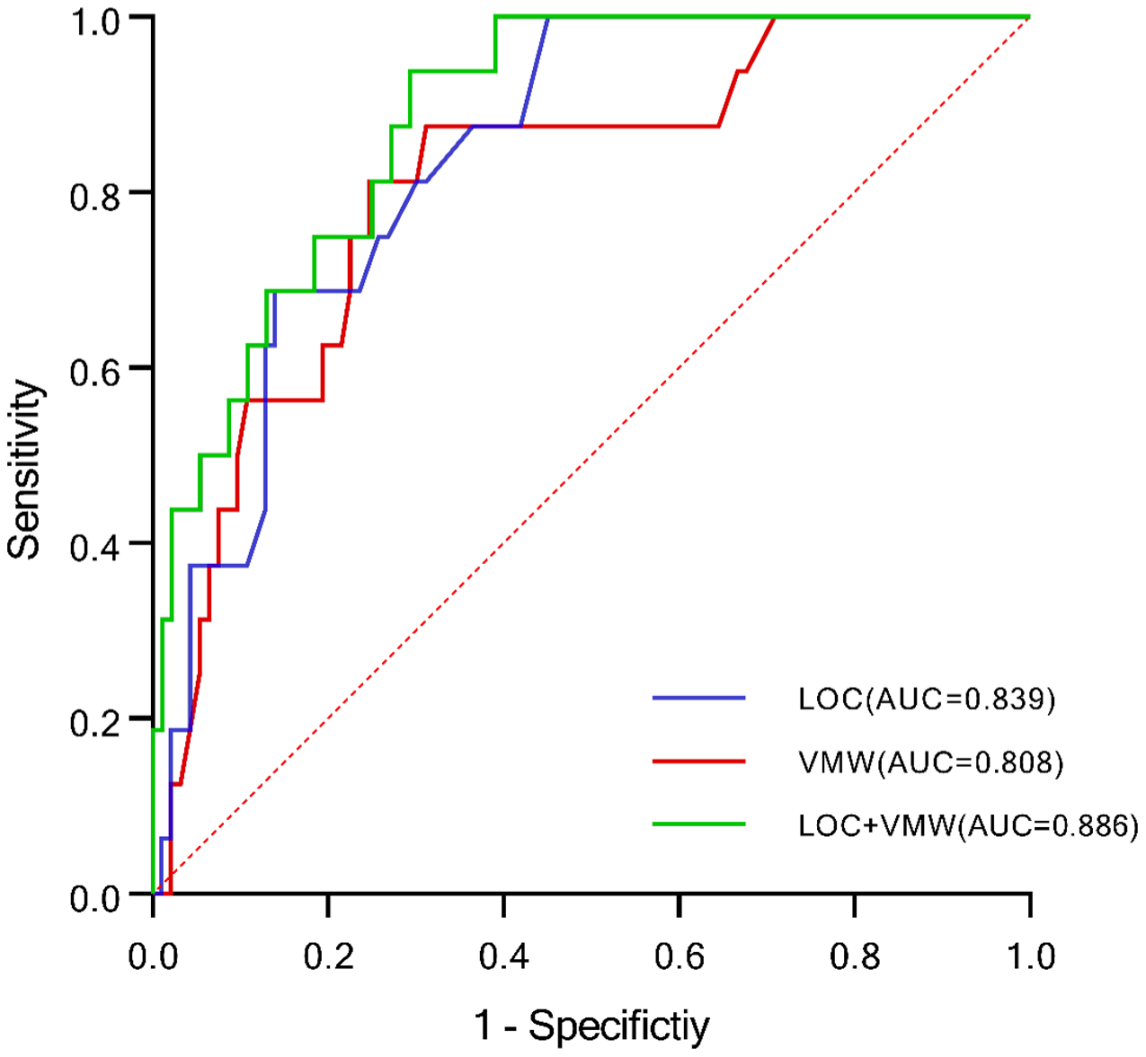
ROC curves demonstrating the diagnostic value of LOC and VMW in DON. LOC, latency onset of constriction; VMW, volume of the medial orbital wall.

Cutoff values in this study were determined using the highest Youden index. ROC curve analysis demonstrated that the AUC for LOC was 0.839 (*p* < 0.001), with a sensitivity of 68.8% and specificity of 86.0%. The cutoff value for LOC was 179.5, providing the highest specificity. For VMW, the AUC was 0.808 (*p* < 0.001), with a sensitivity of 81.3% and specificity of 75.3%. The cutoff value for VMW was −0.065 cm^2^. A multivariate model combining LOC and VMW was developed using regression analysis. This combined model achieved an AUC of 0.886 (*p* < 0.001), with a sensitivity of 90.5% and specificity of 82.4%, representing the highest predictive performance and sensitivity among all models (Figure 4; Table 5).

**Table 5:** ROC curve depicting the diagnostic value of LOC and VMW in DON.

	AUC	*p*-value	95% CI	Youden index	Sensitivity	Specificity	Cut-off
LOC(ms)	0.839	<0.001^***^	(0.754,0.925)	0.548	68.8%	86.0%^c^	179.5
VMW(cm^2^)	0.808	<0.001^***^	(0.698,0.919)	0.566	81.3%	75.3%	−0.065
LOC+ VMW	0.886^a^	<0.001^***^	(0.815,0.958)	0.648	93.8%^b^	71.0%	0.099

a Denotes the highest predictive performance.

b Denotes the highest sensitivity.

c Denotes the highest specificity.

*P < 0.05, **P < 0.01, ***P < 0.001. LOC, Latency onset of constriction; VMW, The volume of the medial orbital wall.

## 4 Discussion

TAO has been extensively documented to cause visual dysfunction, including decreased visual acuity, visual field defects, color vision abnormalities, reduced contrast sensitivity, and VEP abnormalities (14). Prior studies have indicated that in cases of severe asymmetric visual impairment, particularly when TAO progresses to DON, patients often present with a positive RAPD (15). Clinically, RAPD serves as a critical diagnostic marker for DON. However, research in glaucoma has demonstrated that RAPD is detectable only when approximately 25–50% of retinal ganglion cells are unilaterally lost (16). Furthermore, less than 50% of patients with DON, particularly those with severe bilateral symmetric damage, exhibit RAPD (17). The traditional swinging flashlight test for RAPD is also subject to variability due to environmental lighting, stimulus intensity, and examiner expertise (18, 19). To address these limitations, this study employed the RAPDx pupillometer to objectively quantify dynamic pupillary changes, focusing exclusively on direct light reflex indicators to eliminate potential interference from contralateral visual damage (20). This article underscores the critical roles of pupillary light reflex analysis and MRI in diagnosing and evaluating TAO. This study represents the first application of quantitative pupillary light reflex analysis using RAPDx in conjunction with MRI-based measurements of intraorbital tissue relationships to assist in the diagnosis of DON.

This study analyzed and compared pupillary change indicators across varying severities of TAO. Results indicated that in the more severe TAO groups, LOC prolonged, and LMC progressively slowed. Previous research on optic neuritis (19, 21) has similarly shown delayed LOC and LMC in patients compared to healthy individuals, likely due to functional defects in optic nerve fibers that impair the transmission of light stimuli. However, no significant differences were observed in indicators such as AC, AC%, VC, VD, and PD among the groups. Studies on glaucoma and age-related macular degeneration (AMD) have reported significant reductions in AC and AC% without abnormalities in LOC and LMC, suggesting that the transmission speed of light stimulus signals remains intact, while the amplitude of the response is impaired (22, 23). These findings suggest that visual dysfunction in TAO may primarily stem from pathological changes in the optic nerve.

Pupil size is regulated by the sympathetic and parasympathetic nervous systems. The parasympathetic nervous system controls pupil dilation from its minimum state to three-quarters of its original size, with pupil constriction serving as a marker of parasympathetic activity (24). In this study, indicators such as VC, AC, and AC% reflect sympathetic nervous system function, while VR captures the activity of both sympathetic and parasympathetic systems. Hreidarsson and Laurberg (25) proposed that hyperthyroidism may lead to excessive sympathetic activity. However, Serbest Ceylanoglu et al. (26) found no significant differences in dynamic pupillary change indicators between hyperthyroid patients with TAO and controls. This suggests that abnormal pupillary function in TAO is unlikely to result from autonomic nervous system dysfunction.

The pathogenesis of DON remains poorly understood and is thought to involve mechanisms such as orbital apex crowding, optic nerve stretching, ocular ischemia, and neuritis (11). MRI, with its superior soft tissue resolution and detailed imaging capabilities, is a vital tool for diagnosing DON and evaluating treatment outcomes in patients with TAO (13, 27). In TAO, inflammation and hyperplasia of orbital soft tissues exert pressure on the medial and lateral orbital walls, potentially causing bone deformation. The medial orbital wall, composed of thin spongy bone plates of the ethmoid sinus, is particularly vulnerable. Increased intraorbital pressure can induce bone remodeling, resulting in inward bending of the medial wall (28). In some cases, spontaneous fractures of the medial orbital wall have been reported. This study measured areas of indentation and protrusion in the medial and lateral orbital walls, finding that the medial wall area increased with TAO severity, aligning with previous findings.

In healthy individuals, the optic nerve adopts an “S”-shaped configuration within the orbit to facilitate normal eye movements. Posterior pressure on the eyeball leads to proptosis, which stretches the optic nerve. Some studies have reported that in certain patients with DON, orbital apex crowding due to enlarged extraocular muscles is absent. Instead, increased fat content within and outside the muscle cone may result in spontaneous subluxation of the eyeball and traction neuropathy, contributing to visual dysfunction. In this study, LOC exhibited a moderate positive correlation with IONSL and proptosis, demonstrating a linear relationship. These findings suggest that optic nerve stretching may impair the conduction of light stimulus pulses. Additionally, previous studies (29) have indicated that longitudinal stretching of the optic nerve generates inward shear forces within the optic nerve sheath, potentially compressing the optic nerve. Persistent stretching can disrupt vascular function in the posterior ciliary arteries and peripapillary retinal arteries, leading to neurofiber dysfunction and visual impairment. However, some reports suggest that the optic nerve in patients with DON does not reach its elastic limit, making compression unlikely (30). Further investigation is necessary to elucidate the role of optic nerve stretching in DON pathogenesis.

Previous research has established that the enlargement of extraocular muscles plays a pivotal role in visual dysfunction among patients with TAO and is closely linked to the progression of DON. Studies by Starks et al. (31) and Berger et al. (32) highlighted that the superior rectus and levator palpebrae superioris complex can compress the posterior superior quadrant of the eyeball, impairing blood flow and damaging nerve fiber cells, which leads to irregular visual field defects. In the present study, significant enlargement of all four extraocular muscles was observed as TAO severity increased. Unlike previous investigations, this study excluded the levator palpebrae superioris area from the superior rectus muscle measurements. The optic nerve traverses the optic canal through the narrow orbital apex, making this region particularly vulnerable to compression by the medial and lateral rectus muscles. Measuring the angles between these muscles and the optic nerve provides objective evidence of compression. As the medial and lateral rectus muscles enlarge, these angles decrease, and in some DON cases, the muscle bellies may adhere to the optic nerve (33). Although sectional imaging cannot entirely represent the actual anatomical structures, it offers valuable insights into the pathogenesis of visual dysfunction in TAO and DON progression. Previous studies identified the medial rectus muscle as the sole independent predictor of DON among the extraocular muscles. In this study, the increase in the MRA was moderately correlated with delayed LOC.

Multivariate regression analysis further revealed that LOC and VMW are likely independent predictors of DON. A delayed LOC and increased VMW were associated with a heightened likelihood of DON, consistent with findings by Cheng et al. (34) and Chan et al. (35). These two indicators demonstrated robust diagnostic performance, with an AUC of 0.886, sensitivity of 93.8%, and specificity of 71.0%, establishing a reliable basis for clinical diagnosis. Additionally, this study found that in the more severe TAO groups, the LOC delay was significantly prolonged and showed a moderate positive correlation with IONSL and proptosis. This suggests that optic nerve traction contributes to visual dysfunction in patients with TAO. Multivariate binary logistic regression confirmed VMW and LOC as independent risk factors for DON, with both showing strong diagnostic value.

However, several limitations must be acknowledged. First, the sample size of patients with DON was relatively small, introducing potential bias. Despite this, the findings provide a foundation for future studies on pupillary function in patients with TAO. In recent studies, researchers have achieved quantitative analysis of pupillary light reflex using smartphones and artificial intelligence software. The simpler and more cost-effective equipment facilitates large-scale studies on pupillary light reflex measurement in patients with TAO (36, 37). Second, potential errors in MRI measurements may have affected data accuracy. Finally, as this study was cross-sectional, it could not track dynamic changes in pupillary function before and after treatment, an area that warrants further investigation in future research.

## Data Availability

The raw data supporting the conclusions of this article will be made available by the authors, without undue reservation.
